# Desmopressin and the risk of hyponatremia: A population-based cohort study

**DOI:** 10.1371/journal.pmed.1002930

**Published:** 2019-10-21

**Authors:** Michael Fralick, Sebastian Schneeweiss, Christopher J. D. Wallis, Emily H. Jung, Aaron S. Kesselheim

**Affiliations:** 1 Division of Pharmacoepidemiology and Pharmacoeconomics, Program On Regulation, Therapeutics, And Law (PORTAL), Department of Medicine, Brigham and Women's Hospital and Harvard Medical School, Boston, Massachusetts, United States of America; 2 Eliot Phillipson Clinician Scientist Training Program, Department of Medicine, University of Toronto, Toronto, Canada; 3 Division of Urology, Department of Surgery, University of Toronto, Toronto, Ontario; Harvard Medical School, UNITED STATES

## Abstract

**Background:**

Desmopressin was approved by the Food and Drug Administration (FDA) in 1978 for use in diabetes insipidus and bleeding disorders, but it is also prescribed off-label for patients with nocturia. Quantifying the potential risks facing adult patients taking desmopressin has taken on added importance because a new intranasal formulation of desmopressin was approved by the FDA in 2017. Like the old formulation, the main active ingredient is desmopressin acetate, but the new formulation also contains an excipient designed to enhance absorption. Our objective was to quantify the rate of hyponatremia in routine clinical care for patients prescribed the older formulation of desmopressin.

**Methods and findings:**

We conducted a population-based new-user cohort study from 1 February 2006 to 1 February 2017 using a nationwide commercial health plan database. Patients newly prescribed the older formulation of desmopressin were propensity-score (PS)–matched to patients newly prescribed oxybutynin. As a sensitivity analysis, tamsulosin was used as the comparator rather than oxybutynin. The primary outcome was a primary position diagnosis of hyponatremia. Proportional hazard models after 1:1 PS matching were used to estimate hazard ratios (HRs) and 95% confidence intervals (CI). We identified 3,137 adults who were newly prescribed desmopressin and matched them to 3,137 adults who were newly prescribed oxybutynin. Mean age was 70, 55% were male, 13% filled a prescription for a diuretic during the baseline time period, and the mean baseline sodium prior to receiving either study drug was 140 mmol/L (normal: 135–145). The rate of hyponatremia was 146 per 1,000 person-years for adults prescribed desmopressin compared to 11 per 1,000 person-years for adults prescribed oxybutynin, corresponding to a 13-fold higher rate (HR 13.19; 95% CI 6.69, 26.01; *p* < 0.01). When follow-up was truncated at 30 days, a similar increased rate was observed (HR 19.41; 95% CI 7.11, 52.99; *p* < 0.01). A higher rate of hyponatremia was also observed with desmopressin when tamsulosin was the comparator (HR 12.10; 95% CI 6.54, 22.37; *p* < 0.01). Important limitations of our study include unmeasured confounding (for example, over-the-counter medication use, dietary intake), missing data (i.e., only 20% of patients had a baseline serum sodium), and a lack of data on the newer formulation of desmopressin.

**Conclusions:**

Use of an older formulation of desmopressin was associated with a marked increased rate of subsequent hyponatremia compared to use of other medications indicated for lower urinary tract symptoms. Such risks should be clearly communicated to patients prescribed this formulation of desmopressin.

## Author summary

**Why was this study done?:**

- Desmopressin is a medication that is sometimes used for adults who awake multiple times in the night to urinate, even though it was not previously approved for this purpose by the Food and Drug Administration (FDA).
- One potential risk of desmopressin is a low sodium level (also known as hyponatremia), which can be life-threatening.

**What did the researchers do and find?:**

- We conducted a study using a national healthcare database in the US and observed an increased risk of hyponatremia with the older formulation of desmopressin.
- The rate of hyponatremia was 146 per 1,000 person-years with desmopressin compared to approximately 11 per 1,000 person-years among patients who received oxybutynin.

**What do these findings mean?:**

- The potential increased risk of hyponatremia should be considered prior to prescribing this older formulation of desmopressin.
- Further observational study of the new formulation of desmopressin that was approved by the FDA in 2017, which contains the same active ingredient as the older formulation, to assess for the risk of hyponatremia is warranted.

## Introduction

Desmopressin acetate is a synthetic analog of vasopressin that acts within the collecting ducts to increase water reabsorption in the kidneys and reduce urine production [1,2]. It was initially approved by the Food and Drug Administration (FDA) in 1978 for the treatment of diabetes insipidus and certain rare bleeding disorders [3]. Its approved uses were subsequently extended to include primary nocturnal enuresis in children [4], but this indication was removed from the drug label in 2007 after 61 cases of hyponatremia-related seizures were reported to the FDA [1]. The drug label carried a warning for the risk of hyponatremia [5], but a boxed warning was not mandated.

Desmopressin is still prescribed by some physicians for adults with nocturia [6–8]. Nocturia is defined by the International Continence Society as waking from sleep to void one or more times [9]. Nocturia is often multifactorial in etiology and may be due to an underlying sleep disorder, impaired bladder storage (including reduced bladder capacity or bladder overactivity), urinary factors (including global or nocturnal polyuria), or other factors, including medication or alcohol use [10]. Meta-analyses have identified that desmopressin slightly reduces the absolute number of nocturnal voids per night (i.e., about 0.5 fewer nocturnal voids) compared to placebo but not compared to alpha-blockers (for example, tamsulosin) [2,11].

Despite adult nocturia being an off-label indication, this use of desmopressin has been supported by a number of professional associations, including the American Academy of Family Physicians [8,12]. For this indication, a meta-analysis identified that desmopressin use was associated with a 5.1-fold (95% confidence interval [CI] 3.0, 8.8) higher odds of hyponatremia compared to placebo, with approximately 5% of patients prescribed desmopressin experiencing hyponatremia [2]. Since the clinical trials defined hyponatremia as a decrease in serum sodium level identified through laboratory monitoring, this risk may not be clinically relevant. As a result, the severity of hyponatremia reported in clinical trials may be underestimated because the medication can be stopped before the sodium level decreases and patients become symptomatic and seek medical attention.

Quantifying the potential risks facing adult patients taking desmopressin has taken on added importance because in 2017, a new intranasal formulation of desmopressin was approved by the FDA [1]. Like the old formulation, the main active ingredient is desmopressin acetate, but the new formulation also contains an excipient designed to enhance absorption. The primary objective of this study was to assess the rate of hyponatremia in routine clinical care for patients prescribed this older formulation of desmopressin and determine how it compared to the results derived from the clinical trials.

## Methods

### Study population and design

We conducted a population-based, new-user cohort study using the nationwide US commercial insurance claims database Optum© Clinformatics® Data Mart [13]. This database provides deidentified longitudinal, individual-level data on patient demographics, healthcare utilization, medical diagnoses, diagnostic tests, clinical procedures, outpatient laboratory results, and pharmacy dispensing of drugs to over 20 million people in the US.

We compared patients over the age of 50 years who were newly prescribed desmopressin or oxybutynin between February 1, 2006 (the start of available data) and February 1, 2017 (last available data). We only included patients over the age of 50 because younger patients are more likely to receive desmopressin for another indication (for example, diabetes insipidus or bleeding disorders). Oxybutynin was chosen as the comparator because it too is sometimes prescribed to patients with nocturia but is not associated with hyponatremia. Using an active comparator, rather than a nonuser comparator, improves confounding control and study validity [14,15]. The date of the first prescription for desmopressin or oxybutynin was used as the cohort entry date. “New users” of desmopressin or oxybutynin were defined as patients without a prior prescription for either medication in the 180 days prior to the index date. The 180-day time period is the most commonly used for pharmacoepidemiologic studies using US insurance claims databases, and prior studies have demonstrated similar results when a longer time period is used [16,17].

As a sensitivity analysis, we used an alternate comparator to oxybutynin because the clinical indications for oxybutynin and desmopressin only partially overlap. Tamsulosin was selected as the alternate comparator because it is prescribed for benign prostatic hypertrophy, which is a common cause of nocturia, and it is not known to cause hyponatremia [11],

Patients receiving both desmopressin and oxybutynin on the cohort entry date were excluded. To ensure all patients had at least 180 days of baseline data (i.e., data prior to the index date), we excluded those with less than 180 days of baseline data. For example, if a patient’s insurance coverage started September 1 2017, and they received desmopressin on September 2 2017, then they would not be included because we would only have 1 day of available baseline data for this patient. Patients with any of the following characteristics in the 180 days prior to cohort entry were also excluded: recent hospitalization or a diagnosis of malignancy, hyponatremia, diabetes insipidus, hemophilia A, Von Willebrand disease, or end-stage renal disease requiring dialysis. Our study focused on outpatient prescriptions for desmopressin or its comparator rather than inpatient prescribing.

The study was approved by the institutional review board at Brigham and Women’s Hospital, and a valid data licensing agreement to use Optum Clinformatics was in place.

### Cohort follow-up

Follow-up began the day after cohort entry and continued until the end of the study period, end of continuous health coverage enrollment, occurrence of a study outcome, discontinuation of the initial medication or switching to or adding the comparator medication, end of available patient data, 365 days, or death. A medication was considered discontinued if 14 days elapsed after the expiration of the last prescription’s supply without the prescription being refilled.

### Study outcomes

The primary outcome was the rate of hyponatremia (per 1,000 person-years) after being prescribed desmopressin or oxybutynin. While outpatient interactions are typically limited to a single diagnostic code, hospitalizations are associated with a single primary position diagnosis code and multiple secondary position diagnosis codes. For our primary analysis, we assessed hyponatremia as the primary position diagnosis ICD9 or ICD10 code (inpatient or outpatient) following prescription of desmopressin or oxybutynin. As a sensitivity analysis, we restricted the outcome to primary position inpatient codes. Inpatient codes for hyponatremia have been validated previously and have adequate specificity (>99%) and positive predictive value (>90%) but limited sensitivity (<30%) [18]. As a secondary outcome, we assessed the rate of hyponatremia within the 30 days after being prescribed desmopressin or oxybutynin. This analysis was performed because hyponatremia generally occurs soon after starting desmopressin.

### Baseline covariates

During the 180 days preceding cohort entry (i.e., the date desmopressin or oxybutynin was prescribed), we collected data on chronic medical conditions (for example, hypertension, coronary artery disease, stroke), risk factors for hyponatremia (for example, baseline serum sodium, diuretic use, antidepressant use), healthcare utilization (for example, recent hospitalization, emergency room visit), and concomitant medications (for example, antihypertensives, antipsychotics) for each patient.

### Statistical analysis

Propensity-score (PS) matching was used to adjust for baseline characteristics. The probability of being prescribed desmopressin versus oxybutynin or tamsulosin was calculated through a multivariable logistic regression model that contained all baseline covariates with the exception of laboratory values. Laboratory values were not included because these data were only available for about 20% of patients, and a PS cannot be calculated when there are missing covariate data. There were no other missing data. Covariate balance before and after PS matching was assessed using standardized differences. A standardized difference less than of 0.1 indicates adequate balance between groups [19].

The estimated PS was used to match 1:1 new users of desmopressin with new users of oxybutynin or tamsulosin with a caliper size of 0.05 on the propensity scale. Cox proportional hazards models were used to estimate the hazard ratios (HRs) and 95% CIs of the primary outcome. In a sensitivity analysis, we performed a stratified analysis using deciles of the PS. All analyses were performed using Aetion platform version 3.11 and R version 3.5.0. Aetion has previously been validated by accurately repeating a range of previously published studies and by replicating or predicting clinical trial findings [20–22].

## Results

We identified 232,749 adults who satisfied study inclusion and exclusion criteria (S1A Appendix). Of those, 229,612 were newly prescribed oxybutynin and 3,137 were newly prescribed desmopressin. Adults prescribed desmopressin were more likely to be men, to be younger, to have a slightly higher creatinine, to have benign prostatic hyperplasia, and to have filled a prescription for steroids in the preceding 180 days. Adults prescribed oxybutynin were more likely to have a diagnosis of hypertension or obesity and to have filled a prescription for a diuretic, angiotensin-converting enzyme (ACE) inhibitor, or an older generation antihypertensive in the preceding 180 days (S1B Appendix). The majority of the remaining baseline characteristics (including baseline serum sodium) were balanced before PS matching. The median duration of follow-up was 51 days (interquartile range: 42,121) (S1C Appendix).

After 1:1 PS matching, we were able to match 100% of adults prescribed desmopressin. Overall, the mean age was approximately 70 years, 45% were female, and the baseline sodium was identical for the two groups at 140 mmol/L (normal 135–145). The major risk factors for hyponatremia were relatively rare in both groups (for example, diuretic use) (Table 1) and were well-balanced.

**Table 1:** Baseline patient characteristics.

	Unmatched			Propensity score matched		
Baseline Patient Characteristics	Oxybutynin	Desmopressin	SDiff	Oxybutynin	Desmopressin	SDiff
	229,612	3,137		3,137	3,137	
Cohort entry date:			0.045			0.056
—first quarter	60,369 (26.3%)	771 (24.6%)		843 (26.9%)	771 (24.6%)	
—second quarter	51,434 (22.4%)	715 (22.8%)		716 (22.8%)	715 (22.8%)	
—third quarter	57,880 (25.2%)	835 (26.6%)		807 (25.7%)	835 (26.6%)	
—fourth quarter	59,929 (26.1%)	816 (26.0%)		771 (24.6%)	816 (26.0%)	
Sex, female	172,395 (75.1%)	1,415 (45.1%)	0.643	1,409 (44.9%)	1,415 (45.1%)	0.004
Age, mean (SD)	71.79 (13.83)	69.84 (14.28)	0.139	70.03 (13.07)	69.84 (14.28)	0.014
Serum sodium, mean, mmol/L*	140 (2.78)	140 (2.96)	0.012	140 (3.10)	140 (2.96)	0.007
Serum creatinine, mean, mg/dL*	0.95 (0.35)	1.01 (0.34)	0.162	0.97 (0.29)	1.01 (0.34)	0.127
**Comorbidities**						
Chronic kidney disease	12,521 (5.5%)	134 (4.3%)	0.055	134 (4.3%)	134 (4.3%)	0
Hypertension	125,524 (54.7%)	1,518 (48.4%)	0.126	1,491 (47.5%)	1,518 (48.4%)	0.017
Coronary artery disease	31,107 (13.5%)	427 (13.6%)	0.002	442 (14.1%)	427 (13.6%)	0.014
Heart failure	14,492 (6.3%)	143 (4.6%)	0.077	146 (4.7%)	143 (4.6%)	0.005
Ischemic stroke or TIA	9,865 (4.3%)	101 (3.2%)	0.057	106 (3.4%)	101 (3.2%)	0.009
BPH	22,495 (9.8%)	735 (23.4%)	0.373	742 (23.7%)	735 (23.4%)	0.005
**Medications**						
Beta blocker	58,518 (25.5%)	662 (21.1%)	0.104	688 (21.9%)	662 (21.1%)	0.02
Diuretic	45,715 (19.9%)	417 (13.3%)	0.179	412 (13.1%)	417 (13.3%)	0.005
ACEi	61,553 (26.8%)	657 (20.9%)	0.138	694 (22.1%)	657 (20.9%)	0.029
NSAIDS	39,904 (17.4%)	488 (15.6%)	0.049	468 (14.9%)	488 (15.6%)	0.018
Oral steroids	27,085 (11.8%)	563 (17.9%)	0.174	551 (17.6%)	563 (17.9%)	0.01
Antidepressants	76,017 (33.1%)	942 (30.0%)	0.066	946 (30.2%)	942 (30.0%)	0.003
Antibiotics	82,496 (35.9%)	1,194 (38.1%)	0.044	1,191 (38.0%)	1,194 (38.1%)	0.002
**Healthcare Utilization**						
Colonoscopy	9,950 (4.3%)	160 (5.1%)	0.036	166 (5.3%)	160 (5.1%)	0.009
Flu shot	34,317 (14.9%)	450 (14.3%)	0.017	464 (14.8%)	450 (14.3%)	0.013
Family doctor visit	152,959 (66.6%)	2,043 (65.1%)	0.031	2,063 (65.8%)	2,043 (65.1%)	0.013
ER visit	38,729 (16.9%)	462 (14.7%)	0.059	454 (14.5%)	462 (14.7%)	0.007

**Abbreviations:** ACEi, angiotensin-converting enzyme inhibitor; BPH, benign prostatic hypertrophy; ER, emergency room; NSAID, nonsteroidal anti-inflammatory drug; SD, standard deviation; SDiff, standardized difference; TIA, transient ischemic attack.

*Available for approximately 20% of patients.

### Rate of hyponatremia (oxybutynin comparator)

**Fig 1:**
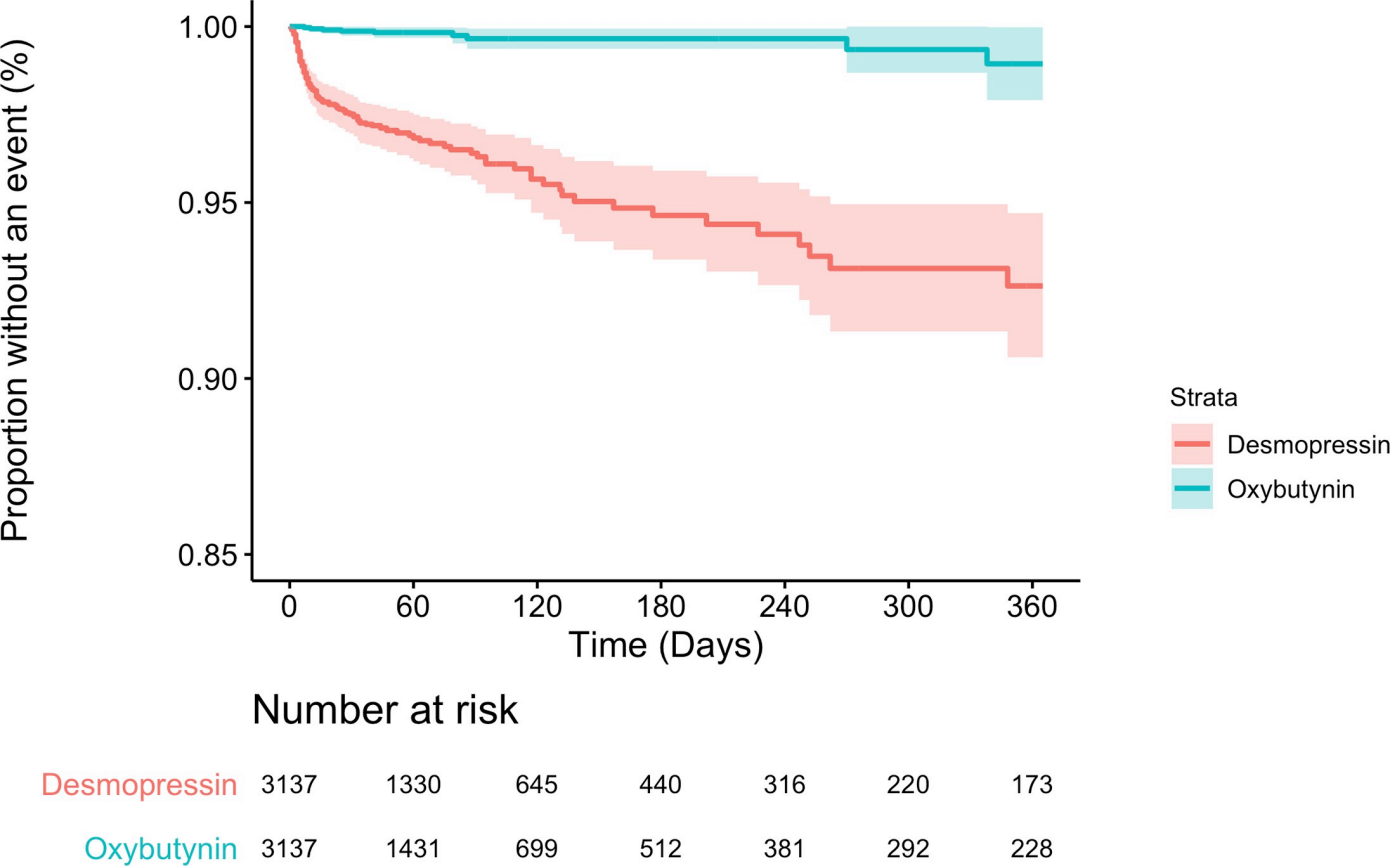
Kaplan–Meier curve for PS-matched rate of hyponatremia. PS, propensity score.

In the unmatched population, there were 114 patients diagnosed with hyponatremia after being prescribed desmopressin (146 events per 1,000 person-years) compared to 836 patients who were prescribed oxybutynin (13 events per 1,000 person-years). This corresponded to an 11-fold higher rate of hyponatremia with desmopressin compared to oxybutynin (HR = 11.42; 95% CI: 9.39, 13.88; *p* < 0.01; Table 2). In the PS-matched population, there were 114 patients who were diagnosed with hyponatremia after being prescribed desmopressin (146 events per 1,000 person-years) compared to nine patients who were prescribed oxybutynin (11 events per 1,000 person-years). This corresponded to a 13-fold increased rate of hyponatremia with desmopressin compared to oxybutynin (HR = 13.19; 95% CI: 6.69, 26.01; *p* < 0.01; Fig 1). Stratifying the PS into deciles provided nearly identical results (HR = 13.06; 95% CI: 10.45,16.32; *p* < 0.01).

**Table 2:** Rate of hyponatremia.

	Unmatched		PS-Matched	
	oxybutynin	desmopressin	oxybutynin	desmopressin
Number of patients	229,612	3,137	3,137	3,137
Number of person-years	66,438	782	850	782
Number of events	836	114	9	114
Rate per 1,000 person-years	12.58	145.78	10.59	145.78
HR (95% CI)	Ref	11.42 (9.39, 13.88), *p* < 0.01	Ref	13.19 (6.69, 26.01), *p* < 0.01

**Abbreviations:** CI, confidence interval; HR, hazard ratio; PS, propensity score; Ref, reference.

Similar results were observed when the rate of hyponatremia within 30 days of receiving either medication was assessed (Table 3). Specifically, 77 patients prescribed desmopressin experienced the outcome (314 per 1,000 person-years) compared to 278 patients prescribed with oxybutynin (15 per 1,000 person-years). After PS matching, this corresponded to a 19-fold increased rate of hyponatremia with desmopressin compared to oxybutynin (HR = 19.41; 95% CI 7.11, 52.99; *p* < 0.01).

**Table 3:** Rate of hyponatremia within 30 days.

	Unmatched		PS-Matched	
	oxybutynin	desmopressin	oxybutynin	desmopressin
Number of patients	229,612	3,137	3,136	3,136
Number of person-years	18,314	245	247	245
Number of events	278	77	4	77
Rate per 1,000 person-years	15.18	313.99	16.21	314.1
30-day risk of hyponatremia	0.12%	2.50%	0.13%	2.50%
HR	Ref	20.71 (16.09, 26.65), *p* < 0.01	Ref	19.41 (7.11, 52.99), *p* < 0.01

**Abbreviations:** HR, hazard ratio; PS, propensity score; Ref, reference.

In our sensitivity analysis that defined hyponatremia using the primary position inpatient diagnosis code alone, 30 patients prescribed desmopressin were hospitalized with hyponatremia (38 per 1,000 person-years) compared to 136 patients prescribed oxybutynin (2 events per 1,000 person-years). After PS matching, this corresponded to a nearly 30-fold higher rate of hyponatremia hospitalization (HR = 29.83; 95% CI 4.11, 216.45; *p* < 0.01).

### Rate of hyponatremia (tamsulosin comparator)

We identified 532,433 adults who were newly prescribed tamsulosin and 3,031 who were newly prescribed desmopressin. Adults prescribed desmopressin were less likely to be male and to have benign prostatic hyperplasia or cardiovascular disease. Adults prescribed tamsulosin were less likely to be prescribed steroids, anxiolytics/antidepressants, or medications for dementia. After PS matching, these characteristics and all other measured characteristics were well balanced.

In the unmatched population, there were 134 patients who were diagnosed with hyponatremia after being prescribed desmopressin (177 events per 1,000 person-years) compared to 1,466 patients after being prescribed tamsulosin (8.3 events per 1,000 person-years). This corresponded to a 20-fold higher rate of hyponatremia with desmopressin compared to tamsulosin (HR = 20.00; 95% CI: 16.74, 23.86; *p* < 0.01). In the PS-matched population, there were 134 patients who were diagnosed with hyponatremia after being prescribed desmopressin (177 events per 1,000 person-years) compared to 11 patients who were prescribed tamsulosin (14.5 events per 1,000 person-years). This corresponded to a 12-fold higher rate of hyponatremia with desmopressin compared to tamsulosin (HR = 12.10; 95% CI: 6.54, 22.37; *p* < 0.01). In our PS-matched sensitivity analysis restricted to 30 days of follow-up, an increased rate of hyponatremia with desmopressin compared to tamsulosin was also apparent (HR = 29.23; 95% CI: 9.31, 91.84; *p* < 0.01).

## Discussion

In this large cohort study of older adults who were newly prescribed desmopressin, we identified an increased rate of hyponatremia diagnosis soon after the medication was prescribed. The findings were robust regardless of the comparator medication used and were also observed using a shorter follow-up period of 30 days. These findings raise concerns about the safety of this formulation of desmopressin, particularly if the medication is being used off-label for the treatment of nocturia.

The increased risk of hyponatremia associated with desmopressin is consistent with published meta-analyses of clinical trials [2]. However, we found that the absolute rate of hyponatremia was approximately 146 per 1,000 person-years, which is many-fold higher than rates reported in the clinical trial literature. One reason for the lower rate observed in clinical trials may be that trial participants have closely monitored serum sodium levels. Frequent laboratory monitoring allows for treatment discontinuation if the sodium level begins to drop and approaches, but does not yet reach, the cutoff for hyponatremia. In addition, the patients included in our real-world cohort were at higher risk for hyponatremia because they were older and had more comorbid conditions than those in the trials included in the meta-analysis [2,11].

An alternate explanation for the higher rate of hyponatremia we observed might be reverse causation because some patients with severe hyponatremia are treated with desmopressin. We think this is unlikely because we excluded patients with a recent diagnosis of hyponatremia and because the baseline serum sodium values for the patients who had laboratory measures were normal. It is possible we may have missed some patients who were hospitalized for another diagnosis but also had hyponatremia during the hospitalization. However, we think this is also unlikely because we excluded all patients with a recent hospitalization within the preceding 180 days.

Another potential limitation of our study is unmeasured confounding. For example, we did not have access to some over-the-counter medications that are associated with hyponatremia (for example, nonsteroidal anti-inflammatory drugs) or other risk factors for hyponatremia (for example, low-solute diet coupled with high water intake, alcohol use). Furthermore, we identified hyponatremia using insurance claims diagnoses, which have acceptable specificity but poor sensitivity. Another limitation is that we assumed censoring was noninformative. While we included a grace period of up to 14 days after treatment discontinuation to identify potential study outcomes during this time period, we may have missed outcomes if, for example, the patient was censored because they died. Finally, an important limitation is that we lacked the sample size to calculate the rate of hyponatremia with various doses of desmopressin.

Quantifying the potential risks facing adult patients taking desmopressin has taken on added importance because, in 2017, a new intranasal formulation of desmopressin was approved by the FDA for the treatment of nocturia due to nocturnal polyuria [1]. The new formulation contains an excipient designed to enhance absorption across the nasal mucosa. In clinical trials of the intranasal formulation of desmopressin, approximately 5% of patients developed mild hyponatremia, and 1% developed severe hyponatremia [1]. Since the new formulation of intranasal desmopressin was only marketed in 2018, data for its use in routine care are not yet available. However, it will be important to investigate outcomes from its clinical use using similar methods described in this study to monitor how the risk of hyponatremia observed in the pivotal clinical trials will compare to the risk in routine practice.

## Conclusions

The observed rate of hyponatremia in the 30 days after initiation of an older formulation of desmopressin of 314 per 1,000 person-years was higher than the reported rate in prior clinical trials. This increased rate of subsequent hyponatremia was observed in comparison to use of other medications indicated for lower urinary tract symptoms. These risks should be clearly communicated to patients prescribed this formulation of desmopressin. This increased rate of hyponatremia may also be relevant to patients considering use of a newer formulation of desmopressin that was recently approved by the FDA for the treatment of nocturnal polyuria. Real-world studies to quantify the risk of hyponatremia with this newer formulation will be necessary to ensure the risks do not outweigh the benefits.

## Supporting information

S1 Appendix Table A: Cohort creation. Table B: Baseline characteristics before and after PS matching. Table C: Reasons for censoring. Table D: Table. STROBE statement. PS, propensity score; STROBE, STrengthening the Reporting of OBservational studies in Epidemiology.
(DOCX)

## Abbreviations

ACE: angiotensin-converting enzyme
CI: confidence interval
FDA: Food and Drug Administration
HR: hazard ratio
PS: propensity score

## References

[1] Fralick M, Kesselheim AS. FDA Approval of Desmopressin for Nocturia. JAMA 2017;317:2095–2060. 10.1001/jama.2017.4316 28384655

[2] Ebell MH, Radke T, Gardner J. A systematic review of the efficacy and safety of desmopressin for nocturia in adults. J Urol. 2014;192(3):829–35. 10.1016/j.juro.2014.03.095 24704009

[3] Menon C, Berry EW, Ockelford P. Beneficial Effect of DDAVP on Bleeding Time Von Willebrand’s Disease. Lancet. 1978;312(8092):743–4. 10.1016/s0140-6736(78)92749-6 80674

[4] Moffatt ME, Harlos S, Kirshen AJ, Burd L. Desmopressin acetate and nocturnal enuresis: how much do we know? Pediatrics. 1993 9;92(3):420–5. 8361796

[5] U.S. Food and Drug Administration. Desmopressin drug label. 2007 [cited 2018 Jul 1]. Available from: https://www.accessdata.fda.gov/drugsatfda_docs/label/2007/017922s038,018938s027,019955s013lbl.pdf.

[6] Lose G, Mattiasson A, Walter S, Lalos O, Kerrebroeck, Van P, Abrams P, et al Clinical experiences with desmopressin for long-term treatment of nocturia. J Urol. 2004;172(3):1021–5. 10.1097/01.ju.0000136203.76320.f6 15311028

[7] Laureanno P, Ellsworth P. Demystifying nocturia: identifying the cause and tailoring the treatment. Urol Nurs. 2010;30(5):276–86. 21067093

[8] Weiss JP, Blaivas JG, Bliwise DL, Dmochowski RR, Dubeau CE, Lowe FC, et al The evaluation and treatment of nocturia: A consensus statement. BJU Int. 2011;108(1):6–21. 10.1111/j.1464-410X.2011.10175.x 21676145

[9] Van Kerrebroeck P, Abrams P, Chaikin D, Donovan J, Fonda D, Jackson S, et al The standardisation of terminology in nocturia: report from the Standardisation Sub-committee of the International Continence Society. Neurourol Urodyn. 2002;21(2):179–83. 11857672 10.1002/nau.10053

[10] Kowalik CG, Cohn JA, Delpe S, Reynolds WS, Kaufman MR, Milam DF, et al Nocturia: Evaluation and Current Management Strategies. Rev Urol. 2018;20(1):1–6. 10.3909/riu0797 29942194 PMC6008257

[11] Han J, Jung JH, Bakker CJ, Ebell MH, Dahm P. Desmopressin for treating nocturia in men. BJU Int. 2018;122(4):549–59. 10.1111/bju.14183 29489052

[12] Slawson D. Desmopressin Effective for Treating Nocturia in Adults. Am Fam Physician. 2014;90(11):796a–797.

[13] Fralick M, Kim SC, Schneeweiss S, Kim D, Redelmeier DA, Patorno E. Fracture risk after initiation of use of canagliflozin: A cohort study. Ann Intern Med. 2019;170(3):155–63. 10.7326/M18-0567 30597484 PMC6602870

[14] Schneeweiss S. A basic study design for expedited safety signal evaluation based on electronic healthcare data. Pharmacoepidemiol Drug Saf. 2010;19(8):858–68. 10.1002/pds.1926 20681003 PMC2917262

[15] Goodman SN, Schneeweiss S, Baiocchi M. Using design thinking to differentiate useful from misleading evidence in observational research. JAMA. 2017;317(7):705–7. 10.1001/jama.2016.19970 28241335

[16] Connolly JG, Schneeweiss S, Glynn RJ, Gagne JJ. Quantifying bias reduction with fixed-duration versus all-available covariate assessment periods. Pharmacoepidemiol Drug Saf. 2019;28(5):665–70. 10.1002/pds.4729 30786103

[17] Fralick M, Sacks CA, Kesselheim AS. Assessment of Use of Combined Dextromethorphan and Quinidine in Patients with Dementia or Parkinson Disease after US Food and Drug Administration Approval for Pseudobulbar Affect. JAMA Intern Med. 2019;179(2):224–30. 10.1001/jamainternmed.2018.6112 30615021 PMC6439654

[18] Movig KLL, Leufkens HGM, Lenderink AW, Egberts AC. Validity of hospital discharge International Classification of Diseases (ICD) codes for identifying patients with hyponatremia. J Clin Epidemiol. 2003;56(6):530–5. 10.1016/s0895-4356(03)00006-4 12873647

[19] Austin PC. Balance diagnostics for comparing the distribution of baseline covariates between treatment groups in propensity-score matched samples. Stat Med. 2009;28(25):3083–107. 10.1002/sim.3697 19757444 PMC3472075

[20] Wang S, Verpillat P, Rassen J, Patrick A, Garry E, Bartels D. Transparency and reproducibility of observational cohort studies using large healthcare databases. Clin Pharm Ther. 2016;99(3):325–32. 10.1002/cpt.329 26690726 PMC7833029

[21] Fralick M, Kesselheim AS, Avorn J, Schneeweiss S. Use of health care databases to support supplemental indications of approved medications. JAMA Intern Med. 2018;178(1):55–63. 10.1001/jamainternmed.2017.3919 29159410 PMC5833514

[22] Kim SC, Solomon DH, Rogers JR, Gale S, Klearman M, Sarsour K, et al Cardiovascular safety of tocilizumab versus tumor necrosis factor inhibitors in patients with rheumatoid arthritis—a multi-database cohort study. Arthritis Rheumatol. 2017;69(6):1154–64. 10.1002/art.40084 28245350 PMC5573926

