# Supplementary material for: Desmopressin and the risk of hyponatremia: A population-based cohort study
Source: PLoS Med. 2019 Oct 21;16(10):e1002930. doi: 10.1371/journal.pmed.1002930 (PMC6802819; doi:10.1371/journal.pmed.1002930)
Supplement: S1 Appendix — Table A: Cohort creation. Table B: Baseline characteristics before and after PS matching. Table C: Reasons for censoring. Table D: Table. STROBE statement. PS, propensity score; STROBE, STrengthening the Reporting of OBservational studies in Epidemiology. (DOCX) [file pmed.1002930.s001.docx]

**Appendix: Desmopressin and the Risk of Hyponatremia: A Population-Based Cohort Study**

**Table of Contents**

Overview of study protocol page 2 – 3
Cohort creation page 4

Complete baseline characteristics page 5 - 7

Reasons for censoring page 8

STROBE Page 9-12

**Study Protocol**

**Date created:** October 10, 2018
**Most recent revision:** July 29, 2019

**Research Question:** Is desmopressin use associated with an increased rate of hyponatremia?

**Study Design:** New-user active comparator cohort study.

**Comparators:** Oxybutynin or Tamsulosin.

**Index date:** The date of cohort entry (i.e., index date) was the date of first prescription for desmopressin or its comparator.

**Data source:** Optum Clinformatics Data Mart.

**Study outcome:** For our primary analysis, we assessed hyponatremia as the primary position diagnosis ICD9 or ICD10 (inpatient or outpatient) following a fill for a prescription for desmopressin or its comparator [ICD9: 276.1, ICD10: E87.1]. As a sensitivity analysis, we restricted the outcome to primary position inpatient codes.

**Inclusion criteria:**

- Newly prescribed oxybutynin or desmopressin from 1 February 2006 to 1 February 2017
  - “new” was defined as not having a prior prescription for either in the preceding 180 days

**Exclusion criteria:**

- Recorded in the 180 days prior to the index date:
  - Age < 50 years
  - Diagnosis of hyponatremia
  - Diagnosis of diabetes insipidus
  - Dialysis
  - Diagnosis of Von Willebrand Disease
  - Diagnosis of hemophilia
  - Diagnosis of cancer
  - Inpatient hospitalization

**Statistical Analysis**

- Propensity score matching (1:1 nearest neighbor matching, caliper of 0.05 on the propensity scale, without replacement)
  - Sensitivity analysis: stratification of the propensity score into deciles rather that 1:1 matching
- Cox proportional hazard model to calculate the hazard ratio and 95% confidence interval

**Revisions to protocol**

- In March of 2018 we received additional data and thus extended our study time period from 1 February 2006 – 1 October 2015 to 1 February 2006 – 1 February 2017.
  - In October 2015, the United States switched from ICD9 to ICD10 and thus we also used ICD 10 codes since we now had data after October 2015.
- In December 2018, we decided to include a sensitivity analysis that stratified on the propensity score
- In January 2019, we decided to also include tamsulosin as a comparator medication based on the advice and input of a newly added co-author who is a Urologist

Table A. Cohort creation

| Exclusion criteria |  | Remaining Patients |
| --- | --- | --- |
|  |  | 571,441 |
| Less than 180 days of baseline data | -74,648 | 496,793 |
| Prior use of oxybutynin | -142,410 | 354,383 |
| Prior use of desmopressin | -32,159 | 322,224 |
| Concurrent use of oxybutynin and desmopressin | -147 | 322,077 |
| Age less than 50 years | -62,357 | 259,720 |
| Diabetes insipidus | -170 | 259,550 |
| Prior hyponatremia | -2,690 | 256,860 |
| Von Willebrand Disease | -180 | 256,680 |
| Hemophilia | -55 | 256,625 |
| Cancer | -23,431 | 233,194 |
| Dialysis | -177 | 233,017 |
| Inpatient hospitalization in preceding 180 days | -94 | 232,923 |
| Lack of follow-up | -174 | 232,923 |
| Final cohort |  | 232,749 |

Table B. Baseline characteristics before and after propensity score matching.

|  | oxybutynin | desmopressin | SD | oxybutynin | desmopressin | SD |
| --- | --- | --- | --- | --- | --- | --- |
|  | N = 229612 | N = 3137 |  | N = 3137 | N = 3137 |  |
| Quarter of Cohort Entry Date |  |  | 0.045 |  |  | 0.056 |
| ...First Quarter | 60,369 (26.3%) | 771 (24.6%) |  | 843 (26.9%) | 771 (24.6%) |  |
| ...Second Quarter | 51,434 (22.4%) | 715 (22.8%) |  | 716 (22.8%) | 715 (22.8%) |  |
| ...Third Quarter | 57,880 (25.2%) | 835 (26.6%) |  | 807 (25.7%) | 835 (26.6%) |  |
| ...Fourth Quarter | 59,929 (26.1%) | 816 (26.0%) |  | 771 (24.6%) | 816 (26.0%) |  |
| Female sex | 172,395 (75.1%) | 1,415 (45.1%) | 0.643 | 1,409 (44.9%) | 1,415 (45.1%) | 0.004 |
| Age, mean (sdev) | 71.79 (13.83) | 69.84 (14.28) | 0.139 | 70.03 (13.07) | 69.84 (14.28) | 0.014 |
| **Comorbidities** |  |  |  |  |  |  |
| Acute kidney injury | 6,830 (3.0%) | 71 (2.3%) | 0.045 | 70 (2.2%) | 71 (2.3%) | 0.002 |
| Acute myocardial infarction | 1,955 (0.9%) | 26 (0.8%) | 0.002 | 23 (0.7%) | 26 (0.8%) | 0.011 |
| CABG or PCI | 6,251 (2.7%) | 97 (3.1%) | 0.022 | 78 (2.5%) | 97 (3.1%) | 0.037 |
| Chronic kidney disease | 12,521 (5.5%) | 134 (4.3%) | 0.055 | 134 (4.3%) | 134 (4.3%) | 0 |
| Delirium | 3,960 (1.7%) | 58 (1.8%) | 0.009 | 55 (1.8%) | 58 (1.8%) | 0.007 |
| Dyslipidemia | 102,218 (44.5%) | 1,321 (42.1%) | 0.049 | 1,335 (42.6%) | 1,321 (42.1%) | 0.009 |
| Heart failure | 14,492 (6.3%) | 143 (4.6%) | 0.077 | 146 (4.7%) | 143 (4.6%) | 0.005 |
| Hypertension | 125,524 (54.7%) | 1,518 (48.4%) | 0.126 | 1,491 (47.5%) | 1,518 (48.4%) | 0.017 |
| Coronary artery disease | 31,107 (13.5%) | 427 (13.6%) | 0.002 | 442 (14.1%) | 427 (13.6%) | 0.014 |
| Cerebral vascular disease | 9,865 (4.3%) | 101 (3.2%) | 0.057 | 106 (3.4%) | 101 (3.2%) | 0.009 |
| Obese or overweight | 23,772 (10.4%) | 218 (6.9%) | 0.121 | 195 (6.2%) | 218 (6.9%) | 0.03 |
| Pneumonia | 6,789 (3.0%) | 71 (2.3%) | 0.044 | 75 (2.4%) | 71 (2.3%) | 0.008 |
| Peripheral vascular disease | 15,288 (6.7%) | 168 (5.4%) | 0.055 | 172 (5.5%) | 168 (5.4%) | 0.006 |
| Smoking | 16,145 (7.0%) | 204 (6.5%) | 0.021 | 208 (6.6%) | 204 (6.5%) | 0.005 |
| Type 2 diabetes mellitus | 57,819 (25.2%) | 661 (21.1%) | 0.098 | 635 (20.2%) | 661 (21.1%) | 0.02 |
| Atrial fibrillation | 11,245 (4.9%) | 170 (5.4%) | 0.024 | 182 (5.8%) | 170 (5.4%) | 0.017 |
| Venous thromboembolism | 5,544 (2.4%) | 63 (2.0%) | 0.028 | 54 (1.7%) | 63 (2.0%) | 0.021 |
| Liver disease | 1,070 (0.5%) | 16 (0.5%) | 0.006 | 18 (0.6%) | 16 (0.5%) | 0.009 |
| Intracranial hemorrhage | 1,111 (0.5%) | 22 (0.7%) | 0.028 | 17 (0.5%) | 22 (0.7%) | 0.02 |
| Epilepsy | 2,302 (1.0%) | 36 (1.1%) | 0.014 | 41 (1.3%) | 36 (1.1%) | 0.014 |
| Multiple sclerosis | 3,298 (1.4%) | 71 (2.3%) | 0.061 | 72 (2.3%) | 71 (2.3%) | 0.002 |
| Benign prostatic hypertrophy | 22,495 (9.8%) | 735 (23.4%) | 0.373 | 742 (23.7%) | 735 (23.4%) | 0.005 |
| Dementia | 5,267 (2.3%) | 49 (1.6%) | 0.053 | 62 (2.0%) | 49 (1.6%) | 0.031 |
| Hyponatremia | 0 (0.0%) | 0 (0.0%) | - | 0 (0.0%) | 0 (0.0%) | - |
| **Medications** |  |  |  |  |  |  |
| Diuretic | 45,715 (19.9%) | 417 (13.3%) | 0.179 | 412 (13.1%) | 417 (13.3%) | 0.005 |
| ACEI | 61,553 (26.8%) | 657 (20.9%) | 0.138 | 694 (22.1%) | 657 (20.9%) | 0.029 |
| Angiotensin receptor blocker | 33,514 (14.6%) | 389 (12.4%) | 0.064 | 414 (13.2%) | 389 (12.4%) | 0.024 |
| NSAIDS | 39,904 (17.4%) | 488 (15.6%) | 0.049 | 468 (14.9%) | 488 (15.6%) | 0.018 |
| Oral steroid | 27,085 (11.8%) | 563 (17.9%) | 0.174 | 551 (17.6%) | 563 (17.9%) | 0.01 |
| Statin | 96,397 (42.0%) | 1,156 (36.9%) | 0.105 | 1,145 (36.5%) | 1,156 (36.9%) | 0.007 |
| Sedating medication | 44,964 (19.6%) | 809 (25.8%) | 0.149 | 824 (26.3%) | 809 (25.8%) | 0.011 |
| Inhalers for COPD/asthma | 39,256 (17.1%) | 572 (18.2%) | 0.03 | 577 (18.4%) | 572 (18.2%) | 0.004 |
| Antiplatelet | 14,866 (6.5%) | 185 (5.9%) | 0.024 | 190 (6.1%) | 185 (5.9%) | 0.007 |
| Antiarrhythmic | 3,017 (1.3%) | 53 (1.7%) | 0.031 | 63 (2.0%) | 53 (1.7%) | 0.024 |
| Anticonvulsant | 35,012 (15.2%) | 479 (15.3%) | 0.001 | 500 (15.9%) | 479 (15.3%) | 0.018 |
| Antidepressant | 76,017 (33.1%) | 942 (30.0%) | 0.066 | 946 (30.2%) | 942 (30.0%) | 0.003 |
| Antiparkinsonian medication | 10,077 (4.4%) | 162 (5.2%) | 0.036 | 167 (5.3%) | 162 (5.2%) | 0.007 |
| Antipsychotic | 9,084 (4.0%) | 169 (5.4%) | 0.068 | 180 (5.7%) | 169 (5.4%) | 0.015 |
| Oral anticoagulant | 14,813 (6.5%) | 186 (5.9%) | 0.022 | 203 (6.5%) | 186 (5.9%) | 0.022 |
| Older generation anti-hypertensive | 10,950 (4.8%) | 212 (6.8%) | 0.085 | 215 (6.9%) | 212 (6.8%) | 0.004 |
| Coxib | 6,271 (2.7%) | 96 (3.1%) | 0.02 | 95 (3.0%) | 96 (3.1%) | 0.002 |
| Calcium channel blocker | 35,899 (15.6%) | 514 (16.4%) | 0.02 | 502 (16.0%) | 514 (16.4%) | 0.01 |
| Beta blocker | 58,518 (25.5%) | 662 (21.1%) | 0.104 | 688 (21.9%) | 662 (21.1%) | 0.02 |
| Antibiotic | 82,496 (35.9%) | 1,194 (38.1%) | 0.044 | 1,191 (38.0%) | 1,194 (38.1%) | 0.002 |
| Dementia medication | 11,280 (4.9%) | 145 (4.6%) | 0.014 | 149 (4.7%) | 145 (4.6%) | 0.006 |
| Levothyroxine | 45,281 (19.7%) | 658 (21.0%) | 0.031 | 669 (21.3%) | 658 (21.0%) | 0.009 |
| Number of meds~, mean sdev | 2.79 (3.01) | 2.81 (2.91) | 0.004 | 2.80 (2.95) | 2.81 (2.91) | 0.004 |
| **Healthcare utilization** |  |  |  |  |  |  |
| Lab tests assessing thyroid function | 59,875 (26.1%) | 852 (27.2%) | 0.025 | 862 (27.5%) | 852 (27.2%) | 0.007 |
| Colonoscopy | 9,950 (4.3%) | 160 (5.1%) | 0.036 | 166 (5.3%) | 160 (5.1%) | 0.009 |
| Fecal Occult Blood Test | 10,368 (4.5%) | 142 (4.5%) | 0.001 | 126 (4.0%) | 142 (4.5%) | 0.025 |
| Flu shot | 34,317 (14.9%) | 450 (14.3%) | 0.017 | 464 (14.8%) | 450 (14.3%) | 0.013 |
| Family doctor visit | 152,959 (66.6%) | 2,043 (65.1%) | 0.031 | 2,063 (65.8%) | 2,043 (65.1%) | 0.013 |
| Outpatient visits~, mean sdev | 0.12 (0.31) | 0.13 (0.31) | 0.031 | 0.13 (0.53) | 0.13 (0.31) | 0.009 |
| ER visit | 38,729 (16.9%) | 462 (14.7%) | 0.059 | 454 (14.5%) | 462 (14.7%) | 0.007 |
| Creatinine or metabolic lab panel | 91,145 (39.7%) | 1,225 (39.1%) | 0.013 | 1,246 (39.7%) | 1,225 (39.1%) | 0.014 |
| Creatinine, mean, sdev | 0.95 (0.35) | 1.01 (0.34) | 0.162 | 0.97 (0.29) | 1.01 (0.34) | 0.127 |
| Missing | 184,370 (80.3%) | 2,438 (77.7%) |  | 2,502 (79.8%) | 2,438 (77.7%) |  |
| Serum sodium, mean, mmol/L | 140.22 (2.78) | 140.25 (2.96) | 0.012 | 140.23 (3.10) | 140.25 (2.96) | 0.007 |
| Missing | 185,781 (80.9%) | 2,444 (77.9%) |  | 2,527 (80.6%) | 2,444 (77.9%) |  |
| Metabolic lab panel, mean (sdev) | 0.55 (0.95) | 0.59 (1.42) | 0.032 | 0.59 (1.17) | 0.59 (1.42) | 0.004 |
| Legend: sd = standardized difference, sdev = standard deviation, CABG = coronary artery bypass graft, PCI = percutaneous coronary intervention, ACEi = angiotensin converting enzyme inhibitor, NSAIDS = non-steroidal anti-inflammatory drugs, sedating medication primary includes benzodiazepines and “Z-drugs” (e.g., zolpidem), COPD = chronic obstructive pulmonary disease, ER = emergency room. ~ in preceding 30 days. | | | | | | |

Table C. Reasons for censoring

|  | Overall | oxybutynin | desmopressin |
| --- | --- | --- | --- |
| Study Outcome | 950 (0.4%) | 836 (0.4%) | 114 (3.6%) |
| Death | 1,068 (0.5%) | 1,059 (0.5%) | 9 (0.3%) |
| Start of comparator | 220 (0.1%) | 146 (0.1%) | 74 (2.4%) |
| End of index exposure | 198,878 (85.4%) | 196,303 (85.5%) | 2,575 (82.1%) |
| Maximum follow-up time* | 17,290 (7.4%) | 17,118 (7.5%) | 172 (5.5%) |
| End of available patient data | 478 (0.2%) | 473 (0.2%) | 5 (0.2%) |
| End of insurance | 13,865 (6.0%) | 13,677 (6.0%) | 188 (6.0%) |

*defined as 365 days

Table D. STROBE

STROBE Statement—checklist of items that should be included in reports of observational studies

|  | | Item No. | | Recommendation | Page  No. | Relevant text from manuscript | |
| --- | --- | --- | --- | --- | --- | --- | --- |
| **Title and abstract** | | 1 | | (*a*) Indicate the study’s design with a commonly used term in the title or the abstract | Abstract |  | |
|  |  |  |  | (*b*) Provide in the abstract an informative and balanced summary of what was done and what was found | Abstract |  | |
| Introduction | | | | | |  | |
| Background/rationale | | 2 | | Explain the scientific background and rationale for the investigation being reported | Introduction (paragraph 1-3) |  | |
| Objectives | | 3 | | State specific objectives, including any prespecified hypotheses | Introduction (paragraph 3) |  | |
| Methods | | | | | |  | |
| Study design | | 4 | | Present key elements of study design early in the paper | Methods (paragraph 1&2) |  | |
| Setting | | 5 | | Describe the setting, locations, and relevant dates, including periods of recruitment, exposure, follow-up, and data collection | Methods (paragraph 1&2) |  | |
| Participants | | 6 | | (*a*) *Cohort study*—Give the eligibility criteria, and the sources and methods of selection of participants. Describe methods of follow-up  *Case-control study*—Give the eligibility criteria, and the sources and methods of case ascertainment and control selection. Give the rationale for the choice of cases and controls  *Cross-sectional study*—Give the eligibility criteria, and the sources and methods of selection of participants | Methods (paragraph 1&2) |  | |
|  |  |  |  | (*b*) *Cohort study*—For matched studies, give matching criteria and number of exposed and unexposed  *Case-control study*—For matched studies, give matching criteria and the number of controls per case | Results (paragraph 1) |  | |
| Variables | | 7 | | Clearly define all outcomes, exposures, predictors, potential confounders, and effect modifiers. Give diagnostic criteria, if applicable | Methods  (Study Outcomes, Paragraph 1 and Baseline Covariates Paragraph 1) |  | |
| Data sources/ measurement | | 8* | | For each variable of interest, give sources of data and details of methods of assessment (measurement). Describe comparability of assessment methods if there is more than one group | Methods  (Study Outcomes, Paragraph 1 and Baseline Covariates Paragraph 1) |  | |
| Bias | | 9 | | Describe any efforts to address potential sources of bias | Methods (Statistical Analysis Paragraph 1&2) |  | |
| Study size | | 10 | | Explain how the study size was arrived at | Results (paragraph 1) |  | |
| Quantitative variables | 11 | | Explain how quantitative variables were handled in the analyses. If applicable, describe which groupings were chosen and why | | Methods (Statistical Analysis Paragraph 1&2) |  |  |
| Statistical methods | 12 | | (*a*) Describe all statistical methods, including those used to control for confounding | | Methods (Statistical Analysis Paragraph 1&2) |  |  |
|  |  |  | (*b*) Describe any methods used to examine subgroups and interactions | | Methods (Statistical Analysis Paragraph 1&2) |  |  |
|  |  |  | (*c*) Explain how missing data were addressed | | Methods (Statistical Analysis Paragraph 1&2) |  |  |
|  |  |  | (*d*) *Cohort study*—If applicable, explain how loss to follow-up was addressed  *Case-control study*—If applicable, explain how matching of cases and controls was addressed  *Cross-sectional study*—If applicable, describe analytical methods taking account of sampling strategy | | Methods (Statistical Analysis Paragraph 1&2 and Appendix) |  |  |
|  |  |  | (*e*) Describe any sensitivity analyses | | Methods (Statistical Analysis Paragraph 1&2) |  |  |
| Results | | | | | | |  |
| Participants | 13* | | (a) Report numbers of individuals at each stage of study—eg numbers potentially eligible, examined for eligibility, confirmed eligible, included in the study, completing follow-up, and analysed | | Appendix |  |  |
|  |  |  | (b) Give reasons for non-participation at each stage | | Appendix |  |  |
|  |  |  | (c) Consider use of a flow diagram | | Appendix |  |  |
| Descriptive data | 14* | | (a) Give characteristics of study participants (eg demographic, clinical, social) and information on exposures and potential confounders | | Results (Paragraph 1&2) |  |  |
|  |  |  | (b) Indicate number of participants with missing data for each variable of interest | | Results (Paragraph 1&2) |  |  |
|  |  |  | (c) *Cohort study*—Summarise follow-up time (eg, average and total amount) | | Results (Paragraph 1) |  |  |
| Outcome data | 15* | | *Cohort study*—Report numbers of outcome events or summary measures over time | | Results (Paragraph 3 – 7) |  |  |
|  |  |  | *Case-control study—*Report numbers in each exposure category, or summary measures of exposure | | *NA* |  |  |
|  |  |  | *Cross-sectional study—*Report numbers of outcome events or summary measures | | NA |  |  |
| Main results | 16 | | (*a*) Give unadjusted estimates and, if applicable, confounder-adjusted estimates and their precision (eg, 95% confidence interval). Make clear which confounders were adjusted for and why they were included | | Results (Paragraph 3 – 7) |  |  |
|  |  |  | (*b*) Report category boundaries when continuous variables were categorized | | n/a |  |  |
|  |  |  | (*c*) If relevant, consider translating estimates of relative risk into absolute risk for a meaningful time period | | n/a |  |  |

| Other analyses | 17 | Report other analyses done—eg analyses of subgroups and interactions, and sensitivity analyses | Results (Paragraph 3 – 7) |  |
| --- | --- | --- | --- | --- |
| Discussion | | | | |
| Key results | 18 | Summarise key results with reference to study objectives | Discussion (Paragraph 1) |  |
| Limitations | 19 | Discuss limitations of the study, taking into account sources of potential bias or imprecision. Discuss both direction and magnitude of any potential bias | Discussion (Paragraph 4) |  |
| Interpretation | 20 | Give a cautious overall interpretation of results considering objectives, limitations, multiplicity of analyses, results from similar studies, and other relevant evidence | Conclusions (Paragraph 1) |  |
| Generalisability | 21 | Discuss the generalisability (external validity) of the study results | Discussion (Paragraph 2) |  |
| Other information | |  | | |
| Funding | 22 | Give the source of funding and the role of the funders for the present study and, if applicable, for the original study on which the present article is based | See PLOS |  |

*Give information separately for cases and controls in case-control studies and, if applicable, for exposed and unexposed groups in cohort and cross-sectional studies.

**Note:** An Explanation and Elaboration article discusses each checklist item and gives methodological background and published examples of transparent reporting. The STROBE checklist is best used in conjunction with this article (freely available on the Web sites of PLoS Medicine at http://www.plosmedicine.org/, Annals of Internal Medicine at http://www.annals.org/, and Epidemiology at http://www.epidem.com/). Information on the STROBE Initiative is available at www.strobe-statement.org.
